# Effect of Right Middle-to-Lower Lobe Fixation on Postoperative Lung Volume after Right Upper Lobectomy

**DOI:** 10.5761/atcs.oa.26-00050

**Published:** 2026-06-09

**Authors:** Sang Yun Song, Kyo Seon Lee, Ju Sik Yun

**Affiliations:** 1 Department of Thoracic and Cardiovascular Surgery, Chonnam National University Hwasun Hospital, Chonnam National University Medical School, Hwasunup, Jeollanamdo, Republic of Korea; 2 Department of Thoracic and Cardiovascular Surgery, Chonnam National University Hospital, Chonnam National University Medical School, Gwangju, Republic of Korea

**Keywords:** lung volume, lung function, lung cancer, lobectomy

## Abstract

**Purpose:**

Cranial migration of the right middle lobe (RML) after right upper lobectomy may cause bronchial angulation and alter postoperative lung volume distribution. We investigated whether RML fixation techniques affect postoperative lobar volume and pulmonary function.

**Methods:**

This retrospective single-center study included 96 patients who underwent right upper lobectomy for non–small cell lung cancer (2009–2020). Patients were classified into 3 groups: no fixation, normal fixation, and sliding (anterior–caudal) fixation of the RML to the right lower lobe (RLL). Lobar volumes were assessed using 3-dimensional computed tomography volumetry, and postoperative pulmonary function tests were analyzed when available.

**Results:**

Both lower lobes showed significant compensatory expansion, with the RLL demonstrating the largest increase. Overall, RML volume did not change significantly. However, RML volume increased in the normal fixation group (+58.9 ± 78.2 mL) but decreased in the sliding-fixation (−29.0 ± 92.6 mL) and no-fixation groups (−16.9 ± 118.9 mL), with significant differences among groups (p <0.001). Postoperative changes in forced vital capacity and forced expiratory volume in 1 second did not differ among groups, and RML volume change was not correlated with pulmonary function.

**Conclusions:**

Normal RML fixation preserved RML volume after right upper lobectomy, but did not improve postoperative pulmonary function. Further studies are warranted.

## Abbreviations

CT
: computed tomography
FEV1
: forced expiratory volume in 1 second
FVC
: forced vital capacity
IPL
: inferior pulmonary ligament
LUL
: left upper lobe
LLL
: left lower lobe
PFT
: pulmonary function test
RLL
: right lower lobe
RML
: right middle lobe
RUL
: right upper lobe
VATS
: video-assisted thoracic surgery

## Introduction

Pulmonary lobectomy is the most commonly performed surgical procedure for lung cancer; it is the standard curative treatment for resectable disease. However, lobectomy may cause various postoperative sequelae, with postoperative pain and dyspnea being the most representative and clinically significant ones. Changes in lung volume and pulmonary function are closely related and have been thoroughly investigated following pulmonary resection.^1,2)^

Although lobectomy theoretically causes a proportional reduction in pulmonary function corresponding to the volume of the resected lung, clinical observations suggest otherwise. Postoperative lung volume typically approaches the predicted postoperative value within 1–6 months and increases to nearly 95% of the preoperative lung volume by approximately 1 year owing to compensatory expansion of the remaining lung.^3)^ The extent and pattern of this response, however, vary depending on the resected lobe.

In humans, postoperative pulmonary remodeling is influenced by an upright posture and the unique anatomical characteristics of the thoracic cage, which cannot be fully delineated by animal models. After right upper lobectomy, the right middle lobe (RML) is particularly prone to cranial and posterior displacement, which may lead to bronchial angulation or kinking.^4)^ Bronchial kinking can cause a wide spectrum of clinical manifestations, ranging from mild symptoms such as persistent cough, breathlessness, and airway inflammation to more severe complications such as atelectasis, pneumonia, and decline in pulmonary function. Surgeons have employed various fixation techniques to secure the RML to the right lower lobe (RLL) to prevent torsion after right upper lobectomy.^5–8)^

Postoperative bronchial angle, lung volume distribution, and pulmonary function changes after right upper lobectomy, as well as the anatomical effects of fixation techniques, have been examined in previous studies. However, the effects of RML fixation on postoperative lobar volume preservation and pulmonary function remain unclear. We hypothesized that minimizing postoperative positional and angular changes in the RML by maintaining its anatomical configuration close to the preoperative state could contribute to RML volume preservation after right upper lobectomy, which in turn potentially influences postoperative pulmonary function. This study aimed to evaluate the effects of RML fixation after right upper lobectomy. Patients were classified according to the fixation strategy; moreover, postoperative lobar lung volume and pulmonary function changes were compared to assess the anatomical and functional impact of fixation.

## Materials and Methods

### Patient selection

Patients who underwent right upper lobectomy for non–small cell lung cancer at Chonnam National University Hospital between January 2009 and May 2020 were included in this study. Patients with pathological stage I or II disease and complete medical records were eligible. All included patients were followed up for at least 2 years after surgery and showed no evidence of recurrence or metastasis during the follow-up period. To minimize patient heterogeneity and exclude factors that might influence postoperative lung volume or pulmonary function, the exclusion criteria were defined as follows: perioperative chemotherapy or radiotherapy; concomitant chest wall or rib resection; bronchoplastic procedures, including sleeve lobectomy, decortication, and tourniquet lobectomy; and moderate-to-severe emphysema as determined using computed tomography (CT) imaging. Moreover, only patients with a complete major fissure were included because the completeness of the interlobar fissure between the right middle and lower lobes markedly influences the expansion and mobility of the remaining lobes.

### Surgical procedure

All procedures were performed with single-lung ventilation. Video-assisted thoracoscopic surgery (VATS) lobectomy was the standard approach, with intraoperative conversion to open thoracotomy when necessary. Complete mediastinal lymphadenectomy was routinely performed, although mediastinal lymph node sampling alone was performed in selected cases based on intraoperative judgment. All surgeries were performed by consensus among 3 attending thoracic surgeons. Preservation of the inferior pulmonary ligament (IPL) was the standard procedure in all cases. No adjunctive procedures such as phrenic nerve crushing, diaphragmatic tenting, or pleural tenting were performed. The RML was completely preserved when the minor fissure was well developed; when it was incomplete or unclear, division between the right upper lobe (RUL) and RML was performed at the surgeon’s discretion. Endo GIA (Medtronic, Minneapolis, MN, USA) staplers were used for fissure division in most cases.

### Fixation technique and group classification

**Fig. 1:**
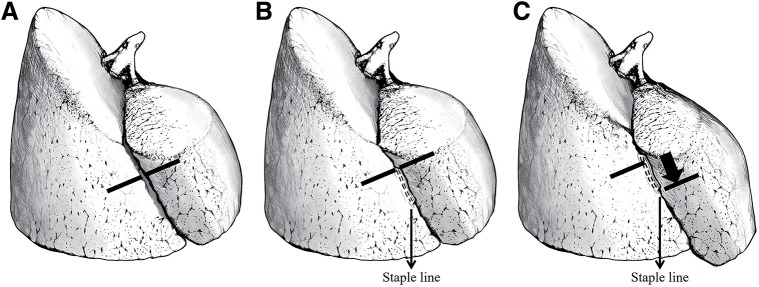
Illustration according to the right middle-to-lower lobe fixation method after right upper lobectomy (thoracoscopic view). (**A**) no fixation, (**B**) normal fixation, and (**C**) sliding fixation. The thick solid line is an imaginary line representing the normal position.

Patients were classified according to the fixation technique. The first group did not undergo fixation (**Fig. 1A**). The second group comprised patients who had the RML fixed to the RLL in a neutral position (normal fixation). In this technique, fixation was performed after right upper lobectomy with full lung expansion while maintaining the remaining lobes in anatomically neutral positions (**Fig. 1B**). The third group comprised patients whose RML was fixed in a modified position (sliding fixation). Here, compared with normal fixation, the RML in this technique was displaced approximately 2–3 cm anteriorly and caudally along the major fissure after lung expansion and before fixation, considering the preoperative anatomical RML position when the RUL was present (**Fig. 1C**). The fixation method varied according to the surgeon and the surgical period. Predominantly, no fixation was performed during the first third of the study period, normal fixation was applied in the middle third, and sliding fixation was employed in the final third. In most cases, fixation was achieved using an Endo GIA stapler; silk or absorbable sutures were used in a small number of patients.

### Postoperative follow-up

Patients with pathological stage I disease underwent chest CT every 6 months after surgery. For patients with pathological stage II disease who exhibited a high risk of early recurrence, chest CT was performed every 3 months during the first 2 postoperative years, followed by surveillance at 6-month intervals. Additional follow-up examinations were performed as indicated.

### Data collection

The collected clinical data included patient age, sex, body mass index, tumor histology, and postoperative pathological stage. Preoperative pulmonary function test (PFT) results were available for all patients, and postoperative results were available for 61 patients. Operative data, including fixation method and surgical approach (VATS or thoracotomy), were also collected and analyzed.

### CT volumetric analysis

Preoperative contrast-enhanced chest CT was performed. Postoperative follow-up chest CT examinations were predominantly conducted using contrast-enhanced CT at appropriate intervals. Whenever possible, CT scans were acquired with a section thickness of ≤1.3 mm to obtain high-quality raw imaging data.

Digital Imaging and Communications in Medicine data were exported from the institutional picture archiving and communication system and transferred to a dedicated workstation equipped with commercially available software (Synapse Vincent 3D-CT Software Analyzer; Fujifilm, Tokyo, Japan). Lung lobe volumes were measured in volume-rendering mode. All volumetric measurements were performed by a single thoracic surgeon with >20 years of experience in thoracic surgery. Initially, lobar segmentation was performed using an automated software algorithm. In cases where the automatic segmentation was suboptimal, manual correction was performed by the examiner to ensure that the lobar boundaries accurately reflected the anatomy.

### Pulmonary function tests

PFTs were performed preoperatively in all patients and approximately 1 year postoperatively. Among the pulmonary function parameters, forced expiratory volume in 1 second (FEV_1_) and forced vital capacity (FVC) were analyzed.

### Statistical analysis

Continuous variables were expressed as mean ± standard deviation. A paired t-test was used to compare the same-patient preoperative and postoperative values. Comparisons between 2 independent groups were performed using the unpaired Student’s t-test or the Mann–Whitney U test, as appropriate, based on data distribution. One-way analysis of variance followed by the Bonferroni post hoc test was applied for intergroup comparisons. Pearson’s correlation coefficient was used to evaluate the linear relationships between continuous variables. All statistical analyses were conducted using SPSS (version 22.0; IBM Corp., Chicago, IL, USA), with a p value <0.05 considered to be statistically significant.

### Ethics statement

The study was conducted in accordance with the principles of the Declaration of Helsinki and its amendments. The study protocol was approved by the Institutional Review Board of the Chonnam National University Hwasun Hospital (IRB Approval No. CNUHH-2026-020). The requirement for informed consent was waived because of the retrospective nature of this study.

## Results

### Patient characteristics and operative data

A total of 96 patients were enrolled, of whom 61 (63.5%) were male. The baseline clinical characteristics of the study population are summarized in **Table 1**. Most patients (87/96, 90.6%) underwent VATS. Seven patients underwent planned thoracotomy, and intraoperative conversion to thoracotomy was required in 2 patients. IPL preservation was achieved in 92 (95.8%) patients; partial IPL division was performed in the remaining 4 to facilitate meticulous dissection of lymph node station #9.

**Table 1:** Clinical characteristics of the patients (n = 96)

Variable	Value
Age (years)	64.0 ± 8.7
Sex, n (%)	
Male	61 (63.5)
Female	35 (36.5)
Body mass index (kg/m^2^)	24.4 ± 3.3
Surgical approach, n (%)	
VATS	87 (90.6)
Thoracotomy	7 (7.3)
Conversion to thoracotomy	2 (2.1)
Lymph node dissection, n (%)	
Mediastinal lymph node dissection	92 (95.8)
Mediastinal lymph node sampling	4 (4.2)
Histology, n (%)	
Adenocarcinoma	78 (81.3)
Squamous cell carcinoma	13 (13.5)
Others	5 (5.2)
Pathologic stage, n (%)	
Stage I	79 (82.3)
Stage II	17 (17.7)
Preoperative pulmonary function	
FEV_1_ (L)	2.57 ± 0.60
FEV_1_ (% predicted)	90.6 ± 13.6
FVC (L)	3.40 ± 0.76
FEV_1_/FVC (%)	76.0 ± 7.8
Preoperative lung volume (mL)	
Right upper lobe	840.8 ± 282.9
RML	449.8 ± 161.0
RLL	1081.1 ± 308.9
Right lung	2371.7 ± 603.9
LUL	1092.7 ± 317.7
LLL	913.5 ± 305.9
Left lung	2006.2 ± 548.0
Fixation group, n (%)	
No-fixation	21 (21.9)
Normal fixation	38 (39.6)
Sliding fixation	37 (38.5)

Values are presented as mean ± standard deviation or number (%).

VATS, video-assisted thoracoscopic surgery; FEV_1_, forced expiratory volume in 1 second; FVC, forced vital capacity; RML, right middle lobe; RLL, right lower lobe; LUL, left upper lobe; LLL, left lower lobe

### Postoperative lung volume changes

**Fig. 2:**
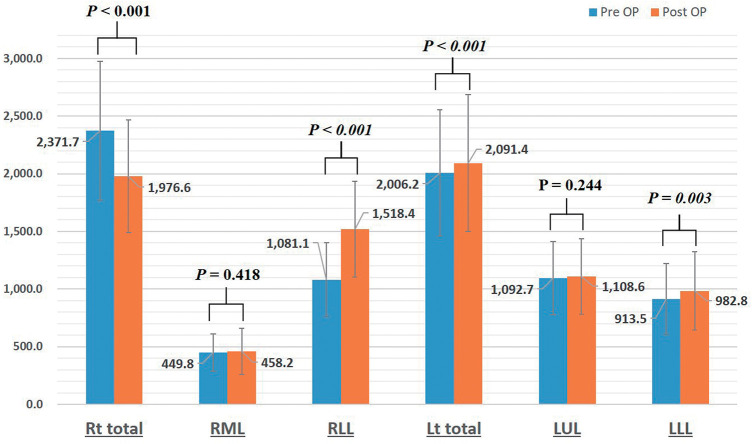
Lung volume changes before and after right upper lobectomy. The mean preoperative and postoperative total lung and individual lobe volumes after right upper lobectomy are shown. Blue bars indicate preoperative volumes, and orange bars indicate postoperative volumes. Lung volumes on the y-axis are expressed in milliliters. p Values denote comparisons between pre- and postoperative measurements. Rt, right; RML, right middle lobe; RLL, right lower lobe; Lt, left; LUL, left upper lobe; LLL, left lower lobe

Pre- and postoperative lung volumes are shown in **Fig. 2**. Mean preoperative RUL volume was 840.8 ± 282.9 mL. Postoperative RML volume increased slightly from 449.0 ± 161.0 mL preoperatively to 458.0 ± 199.3 mL postoperatively; however, this change was not statistically significant. Although the total right lung volume decreased postoperatively, the magnitude of reduction was smaller than that exhibited by the preoperative RUL volume. The RLL demonstrated a significant increase in volume (from 1081.0 ± 319.0 to 1518.0 ± 414.1 mL, p <0.001). In addition, the total left lung volume (from 2006.2 ± 548.0 to 2091.4 ± 593.0 mL, p = 0.003) and the left lower lobe (LLL) volume (from 913.4 ± 305.9 to 982.8 ± 338.7 mL, p <0.001) increased significantly after surgery.

When postoperative lung volume changes were compared between patients who underwent fixation (n = 75) and those who did not (n = 21), no significant differences were observed in the RLL, left upper lobe (LUL), or LLL volume changes. RML volume increased in the fixation group (+15.5 ± 95.9 mL) but decreased in the no-fixation group (–16.9 ± 118.9 mL); however, this difference was not statistically significant (p = 0.085).

**Fig. 3:**
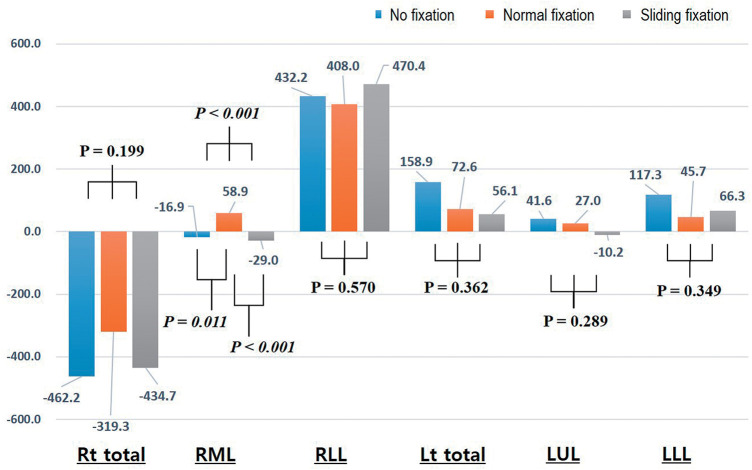
Lung volume changes according to fixation technique after right upper lobectomy. Changes in lung volume after right upper lobectomy according to fixation technique. Patients were categorized into no, normal, and sliding fixation groups. Bars represent the mean changes in total lung and individual lobe volumes. Lung volumes on the y-axis are expressed in milliliters. p Values indicate comparisons among fixation groups. Rt, right; RML, right middle lobe; RLL, right lower lobe; Lt, left; LUL, left upper lobe; LLL, left lower lobe

**Fig. 4:**
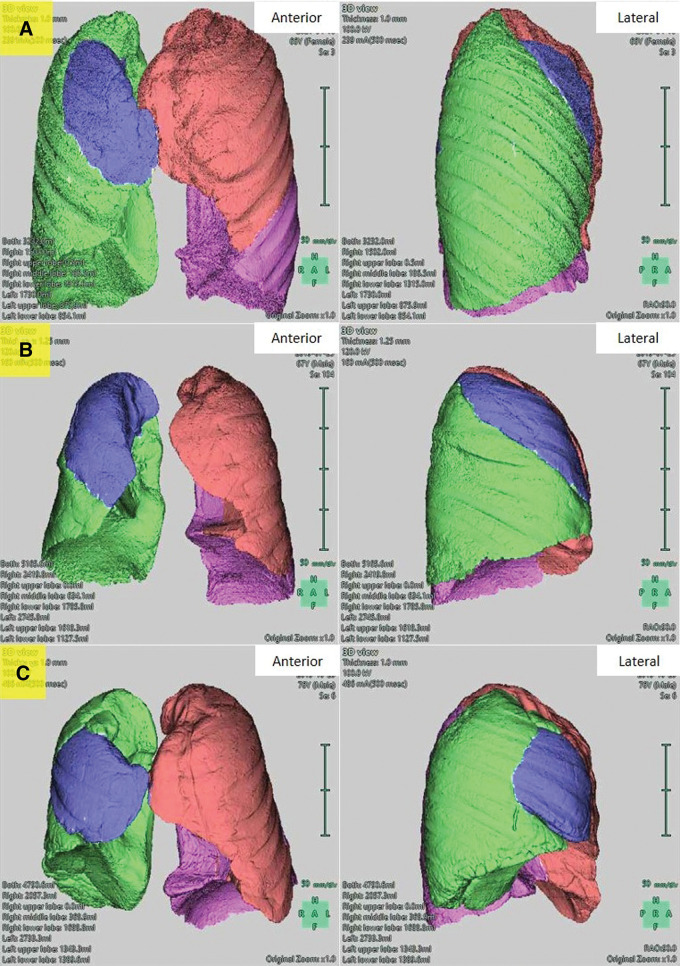
Representative 3-dimensional reconstructions of lung volume changes after right upper lobectomy. Representative 3D volume-rendered images illustrating postoperative lung volume changes after right upper lobectomy according to fixation technique are shown. (**A**) No fixation, demonstrating cranial displacement of the right middle lobe. (**B**) Normal fixation, showing the preservation of the anatomical position of the right middle lobe relative to the RLL. (**C**) Sliding fixation, in which the right middle lobe is repositioned anteriorly and caudally along the major fissure, with relative cranial displacement of the RLL. Images were reconstructed from postoperative chest computed tomography images using 3D volume-rendering software. RLL, right lower lobe

Further comparisons according to fixation technique are shown in **Fig. 3**. RML volume decreased in the no-fixation and sliding fixation groups but increased in the normal fixation group (–16.9 ± 118.9 vs. –29.0 ± 92.6 vs. +58.9 ± 78.2 mL, respectively; p <0.001). No significant differences in the postoperative RLL, LUL, or LLL volume changes were observed between groups. Although the normal fixation group demonstrated a greater increase in RML volume and a smaller reduction in total right lung volume, the difference in the total right lung volume reduction was not statistically significant. Representative 3-dimensional (3D) reconstructions illustrating postoperative morphological differences according to the fixation technique are shown in **Fig. 4**.

### Pulmonary function test outcomes

Postoperative PFT data were available for 61 of the 96 enrolled patients. Postoperative pulmonary function changes according to RML volume change and fixation technique are summarized in **Table 2**. When patients were stratified according to an increase or decrease in RML volume, there were no significant differences in postoperative changes in FVC or FEV₁ between the 2 groups. Similarly, comparison according to fixation technique demonstrated no significant differences in postoperative FVC or FEV₁ changes between the sliding and normal fixation groups.

**Table 2:** Pulmonary function changes according to RML volume change and fixation technique

Factor	FVC change (L)	p Value	FEV_1_ change (L)	p Value
RML volume change				
Increasing (n = 31)	−0.31 ± 0.57	0.946	−0.36 ± 0.46	0.940
Decreasing (n = 30)	−0.32 ± 0.37		−0.37 ± 0.23	
Fixation technique				
Sliding fixation (n = 32)	−0.23 ± 0.41	0.468	−0.33 ± 0.28	0.477
Normal fixation (n = 13)	−0.46 ± 0.72		−0.52 ± 0.59	

Values are presented as mean ± standard deviation.

RML, right middle lobe; FEV_1_, forced expiratory volume in 1 second; FVC, forced vital capacity

**Fig. 5:**
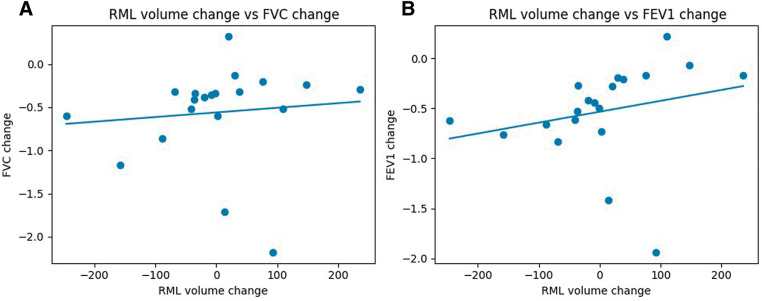
Scatter plots with linear regression lines illustrating the relationship between RML volume change and postoperative pulmonary function changes. (**A**) Volume change versus FVC change (r = −0.033, p = 0.801). (**B**) Volume change versus FEV_1_ change (r = 0.089, p = 0.499). RML volume change was measured in milliliter, and pulmonary function changes were measured in liter. Data points represent individual patients with available postoperative pulmonary function data. FVC, forced vital capacity; FEV_1_, forced expiratory volume in 1 second; RML, right middle lobe

The relationship between RML volume change and postoperative pulmonary function changes is shown in **Fig. 5**. Pearson correlation analysis revealed no significant linear correlation between RML volume change and changes in FVC (r = −0.033, p = 0.801) or FEV_1_ (r = 0.089, p = 0.499).

## Discussion

Pulmonary resection remains an essential treatment option for lung cancer; however, it is associated with a wide range of pulmonary, cardiac, and non-pulmonary postoperative complications. These occur relatively frequently, with reported incidence rates ranging from 9% to 54%, and some can be fatal. These adverse events may significantly affect overall survival and postoperative quality of life.^9,10)^ With advances in minimally invasive surgical techniques, such as VATS and robot-assisted thoracic surgery, postoperative pain and complication incidence have decreased. Nevertheless, a substantial proportion of patients continue to experience chronic postoperative pain or long-term dyspnea after surgery, even in the absence of identifiable causes such as atelectasis, pleural effusion, or overt cardiac dysfunction.^11)^

Multiple factors have been analyzed to address these issues, and various therapeutic approaches have been proposed; however, a fundamental understanding of postoperative anatomical changes and identification of effective mitigation strategies remain essential. Anatomical alterations following pulmonary lobectomy are well recognized. The mechanisms by which the residual thoracic space is filled after lobectomy include overexpansion and displacement of the remaining lung lobes, a mediastinal shift toward the operated side, and elevation of the diaphragm.^12)^

After a right upper lobectomy, the residual thoracic dead space is filled by the displacement and compensatory overexpansion of the remaining lung, which may subsequently induce bronchial deformation or bending. Korst and Humphrey reported that severe post-lobectomy atelectasis is a clinically important postoperative complication, with an incidence of 7.8% among patients undergoing lobectomy. Expansion of the remaining lung after right upper lobectomy may lead to anatomical rearrangement or kinking of the bronchus intermedius or lobar bronchi.^13)^ Yanagihara et al.^14)^ suggested that bronchial kinking after right upper lobectomy is primarily caused by residual lung lobe displacement; they identified significant risk factors for bronchial kinking, including a relatively small RML, a large right thoracic cavity, and a large RUL.

After a right upper lobectomy, cranial transposition of the remaining lower lobe may adversely affect airway remodeling. Although a relatively smaller proportion of the functional lung volume was removed with upper lobectomy than with lower lobectomy (17% ± 4% vs. 27% ± 5%, p <0.001), both anatomical and functional compensation of the residual lung were more pronounced after lower lobectomy (p <0.05).^15)^ Recent reports have also demonstrated that postoperative pulmonary function is superior after lower lobectomy compared with that after upper lobectomy and that the pulmonary reserve of the remaining lobe appears to be more robust following lower lobectomy. Further, these studies showed that the volume of the middle lobe increased after lower lobectomy but decreased after upper lobectomy, which is consistent with the results of previous studies.^16,17)^

Ueda et al. investigated the effect of RML transposition after upper lobectomy on postoperative pulmonary function, where cranial RML migration occurred in 52% of patients. Functional RML volume varied widely, ranging from 9% to 171% of the preoperative value, and these changes were significantly associated with overall pulmonary function. However, no significant differences were observed between patients with and without RML migration in terms of postoperative RML volume, postoperative total lung functional volume, or global pulmonary function.^4)^

Tu et al. reported time-dependent compensatory changes in residual lung lobe volume within 1 year after pulmonary lobectomy. Following right upper lobectomy, all remaining lobes, except the RML, were found to have increased in volume compared with their preoperative values. In addition, a greater rate of volumetric change was observed in the relatively inferior lobe than in the superior lobe of the right lung; specifically, after right upper lobectomy, the RLL demonstrated significantly greater growth than the RML [46.0% (23.4%–80.6%) vs. −4.6% (−25.3%–16.8%), p <0.001]. They further reported that after left lobectomy, the increase in the RLL volume was more pronounced than that in the RUL or RML, and that the increase in the LLL volume exceeded that in the LUL after right lobectomy. These findings are consistent with the results of the present study, in which both lower lobes (RLL and LLL) demonstrated significant postoperative volumetric increases following right upper lobectomy.^3)^ This may be explained by the fact that the lower lobes in humans are subjected to a more direct and sustained influence of diaphragmatic contractions and receive a relatively richer blood supply than the superior lobes.^18)^

Several factors may influence the degree of anatomical deformity after pulmonary lobectomy, including thoracotomy, IPL division, subcarinal lymph node dissection, and negative postoperative pleural suction. Many thoracic surgeons have traditionally believed that IPL division facilitates obliteration of the post-lobectomy dead space by increasing the mobility of the remaining lung lobes. However, several reports have demonstrated that IPL division provides no significant benefit and may instead increase the risk of bronchial stenosis or obstruction as well as postoperative atelectasis; consequently, routine IPL division during upper lobectomy has not been recommended in recent studies.^19,20)^ Moon et al. reported that IPL preservation may be beneficial for LLL expansion and result in less left main bronchus movement and positional changes in patients who underwent LUL.^21)^ Kim et al. reported that IPL division did not confer any significant benefit in terms of RML lung volume or bronchial angle in patients who underwent right upper lobectomy, as assessed by CT; they noted that IPL division was associated with a greater compromise in FVC.^22)^ More recently, Wang et al. reviewed 6 studies that provided the best available evidence to address this clinical question and reported conclusions consistent with those of previous studies.^23)^

One strategy for preventing cranial RML deviation after right upper lobectomy is to fix the RML to the RLL. Traditionally, RML fixation has been introduced to prevent torsion after right upper lobectomy. More recently, fixation has been performed using various techniques with the additional intention of limiting excessive cranial RML displacement and reducing postoperative bronchial angulation. Han et al.^8)^ demonstrated that patients who underwent RML fixation showed significantly less angulation of the right bronchus intermedius compared with those who did not (39.41 ± 9.21° vs. 47.38 ± 10.98°, p = 0.014) and that fixation was effective in preventing postoperative RML atelectasis.

The present study was initiated based on the hypothesis that preventing cranial RML migration after right upper lobectomy and maintaining the anatomical configuration of the bronchus intermedius and RML bronchus as close as possible to the preoperative state might facilitate postoperative RML expansion. We applied an anterior and caudal sliding maneuver to position the RML in the lower anterior thorax and promote RML volume preservation. No significant differences were observed in RLL, LUL, or LLL when postoperative lobar volume changes were compared according to fixation status. In contrast, RML volume increased in patients who underwent fixation, whereas it decreased in those who did not (15.5 vs. −16.9 mL), although this difference did not reach statistical significance (p = 0.085). This lack of significance may be attributable to the relatively small size of the no-fixation group (n = 21); a larger control cohort might have revealed a significant difference.

Further subgroup analysis according to fixation technique demonstrated divergent effects on RML volume. The sliding fixation group exhibited a decrease in RML volume, whereas the normal fixation group showed a significant increase, resulting in a statistically significant difference between the groups (−29.0 vs. +58.9 mL, p <0.001). Contrary to our initial hypothesis, anterior sliding fixation did not lead to increased RML volume. This finding may be explained by the more caudal positioning of the RML, which may have promoted the compensatory cranial expansion of the superior portion of the RLL, thereby limiting RML expansion. Consistent with this interpretation, postoperative 3D reconstructions frequently demonstrated greater cranial expansion of the superior portion of the RLL in patients who underwent sliding fixation (**Fig. 3**). Although the increase in the RLL volume tended to be greater in the sliding fixation group than in the normal fixation group (470.4 vs. 408.0 mL), the difference was not statistically significant.

In contrast to several previous studies,^3,24,25)^ our results did not demonstrate a significant correlation between changes in RML volume and postoperative pulmonary function parameters. This may be explained by the concurrent compensatory expansion of both lower lobes, which likely diminishes the relative contribution of RML volume changes to the overall lung volume and global pulmonary function. The effect of RML volume preservation on postoperative pulmonary function may be attenuated in the context of bilateral lower-lobe compensation. Further studies with larger sample sizes and more comprehensive longitudinal data are warranted to elucidate the relationship between lobar volume changes and functional outcomes.

This study had some limitations. It was a single-center retrospective study, and the sample sizes of the 3 comparison groups were relatively small. In addition, postoperative PFT data were not available for all patients, and the timing of postoperative PFTs was not uniform across the population. Patients with an indistinct minor fissure between the RUL and RML were also not analyzed separately. In patients in whom the minor fissure was unclear and the division between the RUL and RML was performed using a stapler, accurate assessment of anatomical and functional RML volume may have been limited. Finally, postoperative dyspnea was not systematically evaluated or objectively quantified. Given that dyspnea is a clinically relevant outcome that is closely related to postoperative lung volume and functional recovery, future studies that incorporate standardized assessments of patient-reported dyspnea may provide additional insights into the clinical implications of RML fixation.

## Conclusion

No significant overall change in RML volume was observed following right upper lobectomy. The total left lung, LLL, and RLL volumes increased significantly, with the most pronounced increase noted in the RLL. RML volume decreased in patients who underwent sliding fixation and in those who had no fixation, but increased in patients who underwent normal fixation. These volumetric changes were not significantly associated with postoperative pulmonary function. Normal RML fixation may be beneficial for preserving anatomical volume after right upper lobectomy; however, whether this translates into a meaningful improvement in pulmonary function remains unclear. Further studies with larger cohorts and comprehensive clinical assessments are warranted to clarify the functional implications of RML fixation.

## Declarations

### Ethics approval and consent to participate

Not applicable.

### Consent for publication

Written informed consent was obtained from the patient for publication of this report.

### Conflicts of interest/Competing interests

The authors declare no conflict of interest.

### Data Availability

Data that support the findings of this study are available from the corresponding author upon reasonable request.

### Authors’ contributions

Conception and design: Ju Sik Yun, Kyo Seon Lee, Sang Yun Song

(II) Administrative support: Kyo Seon Lee, Sang Yun Song

(III) Provision of study materials or patients: Ju Sik Yun, Sang Yun Song

(IV) Collection and assembly of data: Ju Sik Yun, Kyo Seon Lee

(V) Data analysis and interpretation: Ju Sik Yun, Sang Yun Song

(VI) Manuscript writing: All authors

(VII) Final approval of manuscript: All authors.
